# A novel therapeutic agent, sodium oxybate, improves dystonic symptoms via reduced network-wide activity

**DOI:** 10.1038/s41598-018-34553-x

**Published:** 2018-10-31

**Authors:** Kristina Simonyan, Steven J. Frucht, Andrew Blitzer, Azadeh Hamzehei Sichani, Anna F. Rumbach

**Affiliations:** 1 Department of Otolaryngology, Massachusetts Eye and Ear Infirmary, Harvard Medical School, Boston, MA United States; 2 Department of Neurology, New York University, New York, NY United States; 3 Department of Neurology, Mount Sinai School of Medicine, New York, NY United States; 4 School of Health and Rehabilitation Sciences, Speech Pathology, The University of Queensland, Brisbane, Queensland Australia

## Abstract

Oral medications for the treatment of dystonia are not established. Currently, symptoms of focal dystonia are managed with botulinum toxin injections into the affected muscles. However, the injection effects are short-lived and not beneficial for all patients. We recently reported significant clinical improvement of symptoms with novel investigational oral drug, sodium oxybate, in patients with the alcohol-responsive form of laryngeal focal dystonia. Understanding the mechanism of action of this promising oral agent holds a strong potential for the development of a scientific rationale for its use in dystonia. Therefore, to determine the neural markers of sodium oxybate effects, which may underlie dystonic symptom improvement, we examined brain activity during symptomatic speech production before and after drug intake in patients with laryngeal dystonia and compared to healthy subjects. We found that sodium oxybate significantly attenuated hyperfunctional activity of cerebellar, thalamic and primary/secondary sensorimotor cortical regions. Drug-induced symptom improvement was correlated with decreased-to-normal levels of activity in the right cerebellum. These findings suggest that sodium oxybate shows direct modulatory effects on disorder pathophysiology by acting upon abnormal neural activity within the dystonic network.

## Introduction

Focal dystonia causes abnormal movements of a selective muscle group, often being associated with the production of a specific task. Focal dystonia is the most frequent form of isolated dystonia and may lead to significant occupational disability and life-long social isolation. It becomes even more incapacitating when associated, in about one-third of patients, with dystonic tremor. While the causes of dystonia and dystonic tremor remain unclear, these disorders have been shown to represent heterogeneous conditions at the different ends of the same pathophysiological spectrum^1^. At present, the treatment of focal dystonia with or without tremor is directed to temporary symptom management with botulinum toxin injections into the affected muscles. However, this empirical treatment is beneficial for only a fraction of dystonic patients, showing inconsistent benefits across different forms of dystonia^2–4^. Other pharmacological therapies, particularly the therapeutic options that would modulate the pathophysiology of focal dystonia and dystonic tremor, are not existent.

In search of such therapeutic options, we recently conducted a survey study of 531 patients with the laryngeal form of focal dystonia (LD) with and without tremor, which revealed that dystonic symptoms improve with alcohol ingestion in over 55% of these patients^5^. Alcohol has been shown to modulate cortical gamma-aminobutyric acid (GABA)-ergic neurotransmission^6^, which is abnormal in LD and underlies the pathophysiology of dystonia in general^7–10^. We therefore identified and examined the effects of an oral agent with a therapeutic effect similar to that of alcohol, sodium oxybate (SXB), Xyrem®, in alcohol-responsive (ETOH+) LD patients with and without dystonic tremor of voice (DTv)^11,12^. SXB is the sodium salt of gamma-hydroxybutyric acid (GHB), which, under physiological conditions, is a precursor to GABA^13^. SXB is FDA-approved for the treatment of cataplexy and excessive daytime sleepiness in narcoleptic patients. The Xyrem REMS Program® coordinates and dispenses the drug through a central pharmacy and ensures that prescribers and patients are educated on and understand the risks and safe use conditions, including its very low risk of abuse and misuse^14^. In the initial study, we demonstrated that SXB leads to a significant reduction of voice symptoms in 82.2% of LD and DTv patients^11^.

However, despite this remarkable improvement of dystonic symptoms with an oral medication, the potential central effects of SXB on disorder pathophysiology still remain unclear. This missing information is critical because it undermines the development of a scientific rationale for the potential use of this novel pharmacological agent as a treatment option for alcohol-responsive focal dystonia. Therefore, to identify the mechanisms of SXB central action as well as to determine the combined central effects of SXB and botulinum toxin treatments, we conducted a pharmacological functional MRI (phFMRI) study during symptomatic speech production before and after treatment in two groups of patients with ETOH+ LD and ETOH+ LD/DTv. We hypothesized *a priori* that the beneficial effects of SXB will be due to modulation of abnormal brain activity, particularly within the affected sensorimotor cortical and subcortical circuitry where GABAergic alterations have been described previously^1,10,12,15–18^. We also hypothesized that attenuation of abnormal neural activity by SXB in combination with botulinum toxin treatment would provide further evidence for direct modulation of disorder pathophysiology.

## Results

### The effect of sodium oxybate on abnormal brain activity in ETOH+ drug-responsive patients

**Figure 1:**
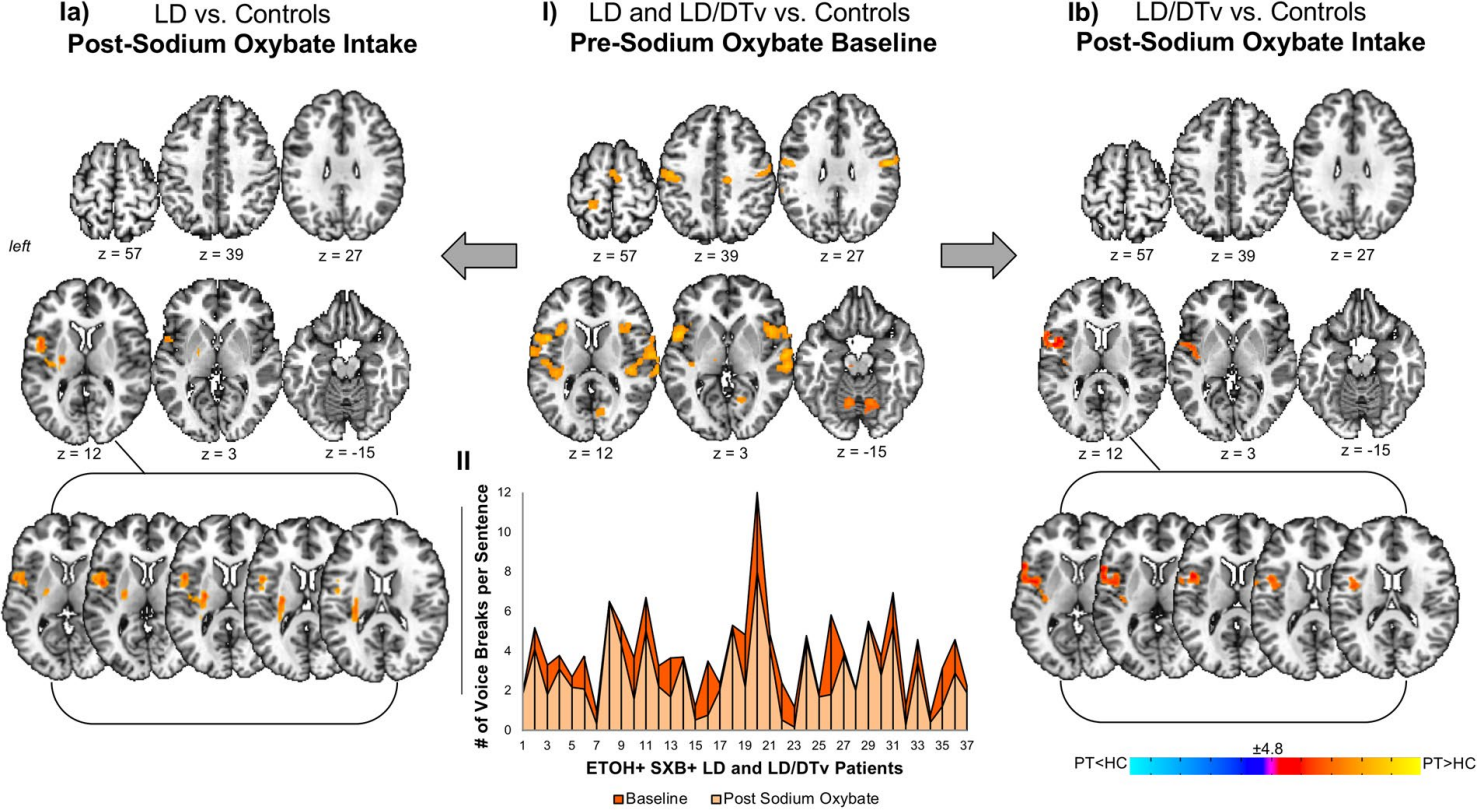
The main effect of sodium oxybate (Xyrem ®) in patients with LD and LD/DTv before and after drug intake compared to healthy subjects. Panel (I) depicts statistically significant differences in brain activation during symptomatic speech production across all patients before drug intake compared to healthy subjects (baseline). Panels (Ia and Ib) show differences in brain activation during symptomatic speech production in LD patients (**Ia**) and LD/DTv patients (**Ib**) after drug intake compared to healthy subjects. Graph (II) shows individual speech symptoms as measured by a number of LD-characteristic voice breaks before and after drug intake in LD and LD/DTv patients. Brain activation differences are shown on a series of axial brain imaging in an AFNI standard Talairach-Tournoux space at FWE-corrected *p* ≤ 0.05.

Similar to the previous studies^1,18^, symptomatic speech production was associated with increased brain activation in bilateral primary sensorimotor, premotor and inferior parietal cortices, insula, parietal operculum, inferior frontal (IFG) and superior temporal (STG) gyri, supplementary motor area (SMA), putamen, thalamus and cerebellum in LD patients both with and without DTv compared to healthy controls (Fig. 1-I). As previously described^11^, SXB intake led to significant reduction of LD and DTv symptoms across all patients (18 LD and 19 LD/DTv). In this study, we found that, abnormal brain activity during speech production was largely attenuated by SXB along with clinical improvement of symptoms in these same patients. That is, there were no statistically significant differences in the majority of functionally abnormal brain regions in patients after drug intake compared to healthy controls at a baseline (Fig. 1-Ia,b). Only few regions remained hyperactive in patients compared to healthy controls, including left parietal operculum and insula in both LD and LD/DTv (Fig. 1-Ia,b) and left putamen in LD (Fig. 1-Ia).

**Figure 2:**
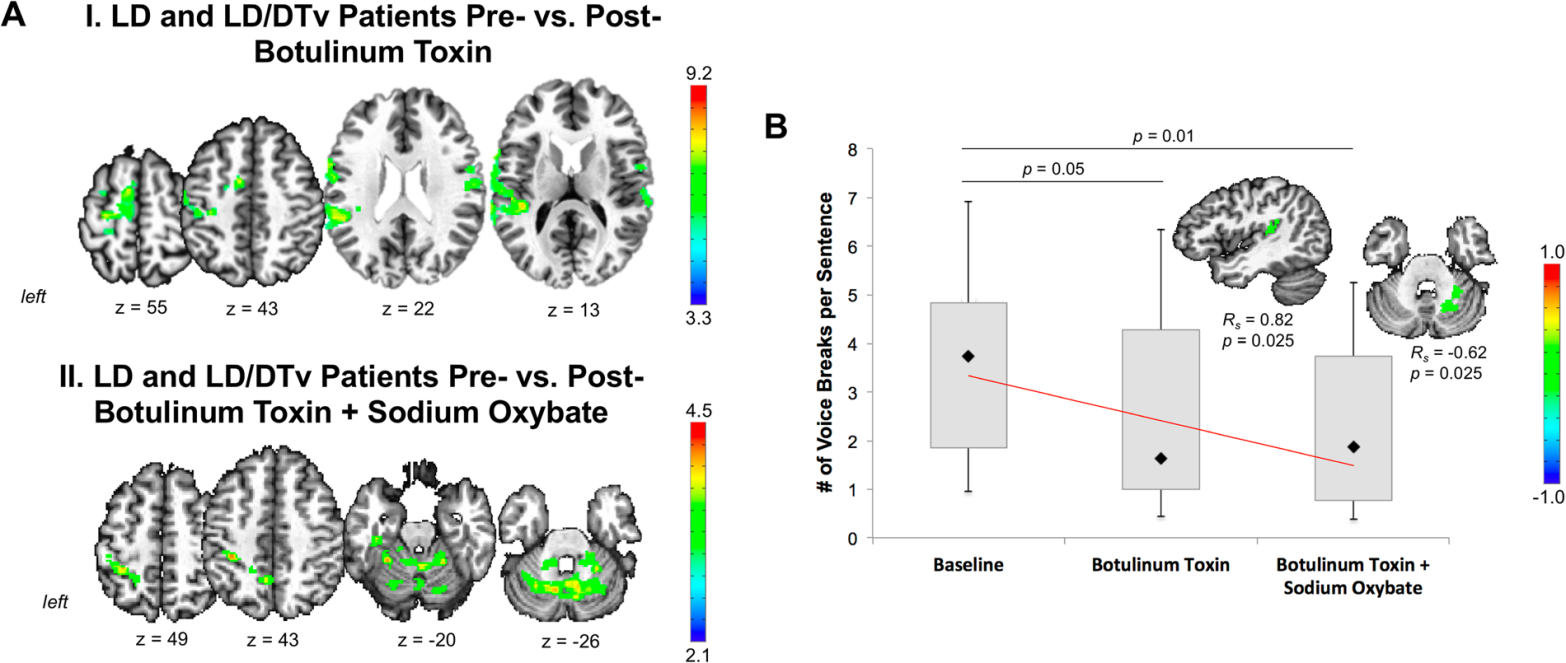
(**A**) Modulatory effect of botulinum toxin alone (I) and in combination with sodium oxybate (II) in patients with LD and LD/DTv. A series of axial brain images depict brain regions that were modulated by the respective treatment in pre vs. post treatment comparisons at FWE-corrected *p* ≤ 0.01. (**B**) A bar chart shows number of voice breaks per sentence in LD and LD/DTv patients at a baseline as well as following botulinum toxin treatment alone and in combination with sodium oxybate. The diamond indicates the median of response. The *p* values indicate the difference between the baseline and post-botulinum toxin assessment as well as between baseline and post-combined botulinum toxin and sodium oxybate assessment. Sagittal and axial brain slices show brain regions where statistically significant relationships were observed between respective treatment and improvement of voice symptoms at a corrected *p* ≤ 0.025. Color bars depict the *t*-level statistics.

When examining the effects of botulinum toxin injections in a subgroup of 13 LD and LD/DTv patients, we found that these patients had a reduction of abnormal brain activity in the left sensorimotor cortex, SMA, and bilateral STG compared to their baseline while symptomatic (Fig. 2A-I). Toxin-induced symptom improvement (based on a number of voice breaks per sentence) was significantly and positively correlated with mean percent BOLD signal change in the left STG (*R*_*s*_ = 0.82, *p* = 0.025) (Fig. 2B).

Administration of SXB to the same LD and LD/DTv patients treated with botulinum toxin showed further improvement of their dystonic voice symptoms^11^ (Fig. 2B) and led to additional attenuation of abnormal activity in the left inferior parietal cortex and bilateral cerebellum (Fig. 2A-II). Right cerebellar (lobule VI) activity showed a significant negative correlation with symptom improvement (based on a number of voice breaks per sentence) following the combined treatment with botulinum toxin and SXB in LD and LD/DTv patients (*R*_*s*_ = −0.62, *p* = 0.025) (Fig. 2B).

### Differential effects of sodium oxybate ETOH+ drug-responsive and drug-nonresponsive patients

**Figure 3:**
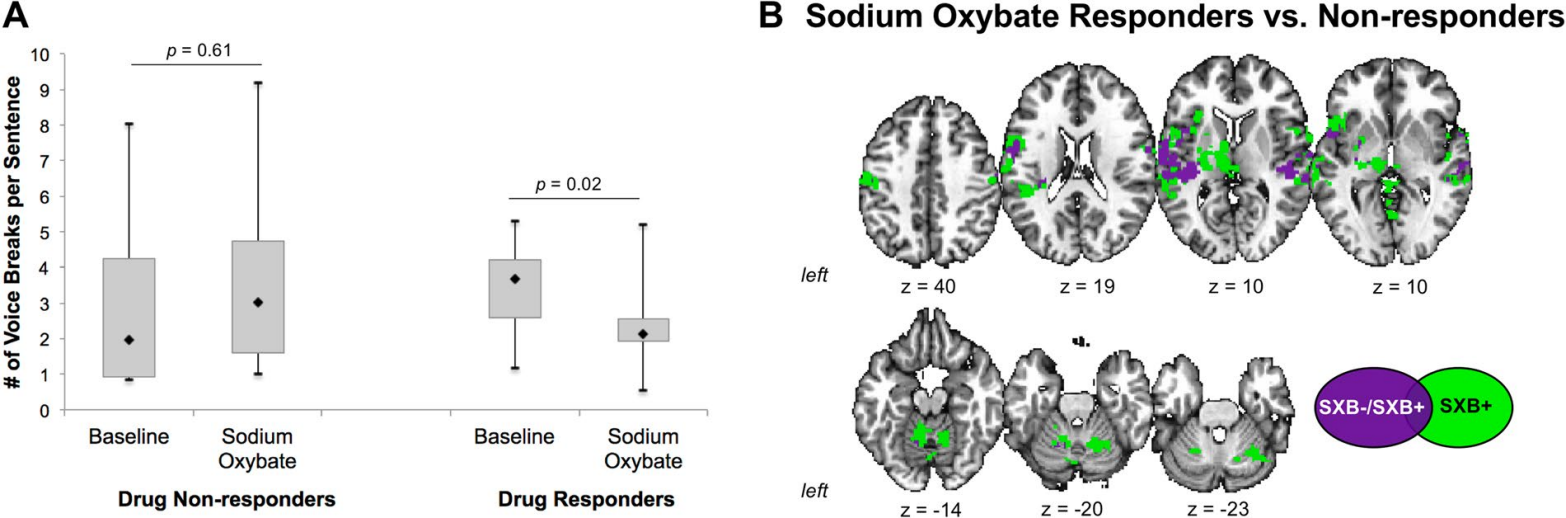
(**A**) A bar chart shows the clinical effects of sodium oxybate in drug-nonresponders and drug-responders, which were assessed based on perceptual analysis of voice symptoms at a baseline and following sodium oxybate treatment. (**B**) A series of axial brain slices show regions of common (in purple) and distinct (in green) action of sodium oxybate in drug-responders and non-responders at FWE-corrected *p* ≤ 0.05.

As we reported previously, while the majority of LD and LD/DTV patients (37 out of 45; 82.2%) had improvement of their symptoms following SXB intake, a smaller portion of patients (8 out of 45; 17.8%) did not show any improvement^11^ (Fig. 3A). Nevertheless, examination of the central drug effects in these 8 SXB- patients vs. 8 age-, gender- and diagnosis-matched SXB+ patients showed a general modulatory effect on abnormal neural activity in the bilateral primary sensorimotor cortex and STG (Fig. 3B). However, in contrast to SXB- patients, the SXB+ group was characterized by much wider distribution of the central drug effects, encompassing larger areas of the primary sensorimotor cortex and STG as well as by additional drug influences on the activity in bilateral IFG, premotor cortex, parietal operculum, inferior parietal cortex, putamen, thalamus, and cerebellum (Fig. 3B). It is important to note that there were no significant differences in brain activation between drug-responsive and drug-nonresponsive patients at their baseline prior to SXB treatment.

## Discussion

We recently demonstrated that SXB significantly reduces dystonic symptoms in the majority of patients with the ETOH+ laryngeal form of isolated focal dystonia^11^. In this study, we outline potential neural influences, by which SXB may exert its action to attenuate abnormal activity within the dystonic cascade, likely underlying clinical improvement of dystonic symptoms.

In line with the assumption that LD and other forms of focal dystonia represent disorders of large-scale functional networks^19–21^, the neuromodulatory effects of SXB were also observed not only on a particular brain region but within the dystonic network, including primary and secondary sensorimotor cortex, IFG, STG, SMA, thalamus and cerebellum. Among these, the cerebellum was the only structure to show a direct relationship between reduced voice symptoms and SXB-attenuated abnormal brain activity. The discussion about the contributing role of cerebellum to the pathophysiology of dystonia has been revived in the past several years as both structural and functional cerebellar abnormalities were consistently described in LD and other forms of dystonia^1,17,20–25^. The imaging findings were further corroborated by neuropathological studies, which demonstrated subtle cerebellar changes in patients with dystonia, including LD^23,26,27^. The cerebellum is positioned uniquely in its access, via thalamus, to primary motor cortex, which is responsible for the final cortical output of dystonic movements. The cerebellum also establishes a two-way neuroanatomical communication with the basal ganglia^28,29^, another prominently abnormal structure within the dystonic cascade, and both regions target the primary motor cortex with an equal number of cerebellar and basal ganglia output neurons via the multisynaptic loops^30,31^. As such, the cerebellum and basal ganglia exert a significant influence on each other’s function as well as on the function of the primary motor cortex. Due to brain network disorganization in dystonia involving these brain regions^19^, cerebellar/basal ganglia influences on motocortical activity become vulnerable and prone to a breakdown. Converging knowledge from the studies in patients with different forms of dystonia suggests that cerebellar dysfunction contributes to abnormal sensorimotor integration or maladaptive plasticity^9^ due, in part, to reduced direct cerebellar modulation of motocortical and basal ganglia excitability^32,33^ and their integrity within the functional network^19^.

To that end, loss of inhibition via a loss of GABAergic function in the sensorimotor cortex and cerebellum^8,10,34^ is considered one of the hallmarks of dystonia pathophysiology^35^. On the other hand, this deficiency may also become a powerful target for the development of novel therapeutic options, such as SXB. Therefore, one possible mechanism of a novel drug action may be based on direct attenuation of cerebellar dysfunction, which triggers a reduction of abnormal activity in the thalamus (direct cerebellar output structure), further propagating to the motor cortex. In line with this, we demonstrated that these regions no longer show abnormal activity following SXB administration in both LD and LD/DTv patients. This finding was further substantiated by the observation of the drug’s effect on brain activity in drug-responsive vs. drug-non-responsive patients. In the former group, symptom improvement was again associated with greater drug-induced modulation of abnormal function in the cerebellum, which potentially cascaded via thalamus and basal ganglia to modulate cortical functional alteration. Given the fact that SXB has been described to act predominantly on GABA_B_^13,36^ and GHB receptors that bind to extrasynaptic GABA_A_ receptors at highest density in thalamus and cerebellum^37^, cerebellar dysfunction might have been one of the major initial targets of SXB action.

A recent study in a large series of patients with different forms of isolated dystonia reported that only 56.6% of LD patients receive botulinum toxin treatment^4^, implying that nearly a half of LD population remain untreated. Symptoms of DTv are known to respond even less well to botulinum toxin^38^. As we demonstrated earlier^11^, SXB improves LD and LD/DTv symptoms on average by 33.5% in 82.2% of our patient cohort, which is an outcome superior to 25.1% symptom improvement with botulinum toxin in only 28.8% of the same cohort. The current study shows that the major neural distinction between botulinum toxin and SXB treatments in LD and LD/DTv may be based on only limited and potentially compensatory effects of botulinum toxin on brain activity, potentially due to peripheral changes in the laryngeal muscle physiology and associated sensorimotor feedback loops, whereas SXB appears to more globally influence the dystonic neural circuitry.

Limitation of this study should be acknowledged. It is important to note that SXB does not completely eliminate LD and LD/DTv symptoms, and its effects last 3.5–4 h. In addition to drug’s pharmacokinetics, a potential explanation to this lay in remaining abnormal putaminal activity within the dystonic network following treatment. As basal ganglia exert modulatory influences on multiple cortical regions^30,31^, we further observed residual increases of functional activity in the cortical regions of sensorimotor integration, namely left parietal operculum and insula, following SXB intake.

As it is the case with any open-label study, a possibility for the placebo effect should also be taken into account. That said, the reproducible kinetics of SXB action in the vast majority of patients (82.2% of patients had symptom improvement) and its central effects on brain regions within the previously established dystonic pathophysiological network may suggest that these therapeutic benefits are not exclusively placebo-driven and likely rooted in direct increases of deficient GABA levels. The latter leads to stabilization of the balance between abnormal excitation and inhibition, resulting in the overall reduction of dystonic symptoms. Thus, examination of the central drug effects of SXB and identification of its potential to modulate the disorder pathophysiology, even in this open-label setting, provided critical initial clues for its impending use in patients with isolated dystonia. These findings have also provided a scientific rationale for continuous investigations of this drug effects that are currently being conducted in a double-blinded placebo-controlled randomized cross-over study.

In summary, we provide first experimental evidence that SXB administration in ETOH+ LD and LD/DTv patients results in attenuation of abnormal brain activity. These effects were seen in the cerebellum, thalamus and sensorimotor cortical regions, but left alterations in the putamen relatively out of the range of SXB’s action. Overall, our findings indicate that SXB may represent a novel pharmacological agent for treatment of LD and LD/DTv symptoms by selectively affecting the pathophysiology of this disorder that pertains to abnormal GABAergic neurotransmission. Taking into account that, among all forms of dystonia, LD has the highest prevalence for no medication use^4^, identification of an oral agent that selectively targets the pathophysiology of this disorder represents an important step towards better clinical management of dystonia.

## Methods

### Study participants

A total of 83 subjects were recruited for this study, including 28 with LD, 25 with LD/DTv, and 30 healthy controls. Four LD and four LD/DTv patients were excluded because of technical problems with image acquisition and the presence of extensive motion artifacts. The final cohorts comprised 24 LD patients (16 females/8 males, 49.7 ± 11.6 years old), 21 LD/DTv patients (18 females/3 males, 56.2 ± 13.2 years old), and 30 healthy controls (19 females/11 males, 50.4 ± 10.8 years old) (see subject detailed demographics in Table 1). None had any past or present history of neurological (except for LD and DTv in the patient groups), psychiatric, or laryngeal problems. Subjects did not differ statistically with respect to their age, gender, disorder onset, or duration (all *p*_uncorr_ ≥ 0.08). All subjects were right-handed, native English-speaking monolingual individuals. Neuroradiological evaluation showed normal brain structure in all subjects without any gross abnormalities. None were carriers of *TOR1A*/DYT1, *THAP1*/DYT6, *TUBB4A*/DYT4, or *GNAL*/DYT25 dystonia mutation as confirmed by genetic screening.

**Table 1:** Subject demographics.

	LD	LD/DTv	HC	*p*-value
**Number of subjects**	24	21	30	
**Age** years; mean ± st. dev.	49.7 ± 11.6	56.2 ± 13.2	50.4 ± 10.8	>0.08
**Gender**	16 F/8 M	18 F/3 M	19 F/11 M	>0.08
**Handedness** Edinburgh Inventory	Right			
**Language**	Monolingual native English			
**Dystonia subtype**	15 ADLD 9ABLD	14 ADLD/DTv 7 ABLD/DTv	N/A	0.78
**Dystonia genetic status**	Negative for DYT1, DYT6, DYT4, DYT25			
**Onset** years; mean ± st. dev.	37.8	42.9	N/A	0.18
**Duration** years; mean ± st. dev.	12.0 ± 10.1	13.3 ± 11.6	N/A	0.67
**Symptom severity** voice breaks per sentence; mean ± st. dev.	3.49 ± 1.92	4.02 ± 2.65	N/A	0.45

Comparisons were made between each patient group and controls as well as between the two patient groups using two-sample *t*-test at a *p* ≤ 0.05. LD – laryngeal dystonia, ADLD – adductor laryngeal dystonia, ABLD – abductor laryngeal dystonia, DTv – dystonic tremor of voice, F – female, M – male, st. dev. – standard deviation, N/A – not applicable.

All patients had only focal form of LD that is their dystonic symptoms were confined to the laryngeal muscles (adductor or abductor, depending of LD phenotype); none had any past or present history of other forms of dystonia affecting other body region. In LD/DTv patients, DTv was localized to the larynx. The LD group included 15 patients with the adductor forms of LD (ADLD) and 9 patients with the abductor form of LD (ABLD) (disorder duration 12.0 ± 10.1 years; average onset at 37.8 years of age). The LD/DTv group included 14 ADLD/DTv patients and 7 ABLD/DTv patients (disorder duration 13.3 ± 11.6 years; average onset at 42.9 years of age). The diagnosis was confirmed by combined examination of medical history, perceptual voice and speech analysis, and fiberoptic nasolaryngoscopy in all patients. All patients were ETOH+ and reported symptom improvement following at least one drink (empirically a 12-oz can of beer, 6-oz glass of wine, or 1-oz shot of hard alcohol).

All subjects provided written informed consent prior to the study participation, which was approved by the Institutional Review Boards (IRB) of the Massachusetts Eye and Ear Infirmary. The drug was administered under the Investigational New Drug protocol (#11,7954, date of registration July 31^st^, 2013) approved by the Food and Drug Administration (NCT01961297). All experiments were performed in accordance with relevant guidelines and regulations.

### Study procedure

All subjects underwent baseline assessments of vital functions, cognitive status, including the Mini-Mental State Examination (MMSE), Montreal Cognitive assessment (MoCA), drug-induced suicidal potential using the Columbia-Suicide Severity Rating Scale (C-SSRS), and daytime sleepiness using the Epworth Sleepiness Scale. Patients receiving botulinum toxin injections participated in the baseline study at least three months after their last injection when fully symptomatic.

All MRI scans were performed on a 3T Philips scanner equipped with an 8-channel head coil. All subjects participated in a baseline fMRI study (i.e., before drug intake). To minimize head movements during scanning, the subjects’ head was tightly cushioned, and they were instructed to remain motionless throughout the scan; all movements were monitored online. High-order shimming was performed before echo planar imaging (EPI) acquisition to minimize EPI distortions and ensure homogeneity of the magnetic field within the scanner. Whole-brain imaging data were obtained using a gradient-weighted EPI pulse sequence (sparse-sampling event-related design: TR = 2 s per volume, 10.6 s between volumes, TE = 30 ms, FA = 90°, FOV = 240 mm, voxel size = 3.75 × 3.75 mm, 36 slices, 4-mm slice thickness) with a blood oxygen level dependent (BOLD) contrast. Subjects were instructed to repeat different symptom-provoking sentences (a total of 32 sentences during four functional runs), which were randomized and acoustically presented one at a time. A structural high-resolution T1-weighted MRI was acquired using 3D magnetization prepared rapid acquisition gradient echo sequence (3D-MPRAGE: TR = 7.5 ms, TE = 3.4 ms, TI = 819 ms, FA = 8°, FOV = 210 mm, 172 slices, 1-mm slice thickness) as an anatomical reference in functional data analyses. Imaging data in healthy controls were used to examine differences between patient and healthy groups at a baseline.

After completion of the baseline scan, all patients were administered an oral dose of SXB, as described previously^11^. Briefly, patients who reported alcohol-induced symptom improvement after one drink received 1.0 g of SXB (14 LD and 16 LD/DTv); patients who reported symptom improvement after two or more drinks received 1.5 g of SXB (10 LD and 5 LD/DTv). The decision regarding the dosing was made based on a combination of our prior clinical experience in improving dystonic symptoms with alcohol and sodium oxybate^12,39–41^ and each patient’s alcohol tolerance^11^. Transient minor side effects were observed in 14 LD and 11 LD/DTv patients and included slight lightheadedness, drowsiness, dizziness, or headache. All side effects resolved within 45–60 min following drug intake^11^. All clinical assessments described above at a baseline were repeated 40 min following drug intake; post-drug MRI was performed at 60 min. Patients were discharged after the completion of study procedures 5 hours after drug intake.

Only 13 patients (6 LD and 7 LD/DTv) have been receiving botulinum toxin injections on a regular basis for the management of their symptoms. These patients returned for the follow-up study at the peak of their botulinum toxin treatment (1.5 months after the last injection) to examine the combined effects of SXB and botulinum toxin. The same procedures as described above were repeated for these patients.

As reported earlier^11^, 18 LD and 19 LD/DTv had a clinical response to SXB (from here on SXB+), whereas 6 LD and 2 LD/DTv patients did not have any symptom improvement (from here on SXB-). This assessment was based on the perceptual evaluation of voice symptoms, which were audio recorded at a baseline and 40 min after drug intake using 20 sentences with high content of vowels to elicit ADSD symptoms and 20 sentences with high content of voiceless consonants to elicit ABSD symptoms^38^. Voice recordings were anonymized and randomized for pre- and post-drug assessments; voice symptoms (breaks, harshness, breathiness and tremor) were blindly rated by an experienced speech-language pathologist (AFR), as reported previously^1,12,18,22,23,42^. There was no significant relationship between the symptom severity and treatment response (SD: all *r* ≤ 0.16, all *p* ≥ 0.46; SD/DTv: all *r* ≤ 0.21, all *p* ≥ 0.35).

The primary aim of this study was to examine the central effects of drug benefits in SXB+ patients. As a secondary exploratory aim, we examined central drug effects in SXB+ and SXB− patients in order to investigate the putative pathways that are selectively modulated in drug-responders vs. drug-nonresponders.

### MRI analysis

Imaging data were analyzed using AFNI and FSL software. Following standard pre-processing, the first four volumes in each subject’s time series were discarded to account for magnetization equilibrium. All images were visually inspected for motion artifacts. To assess head motion in each subject, the root mean square (RMS) was calculated for each individual across the entire phfMRI session. Two-sample *t-*tests between the groups showed no significant differences in head motion at *p* ≥ 0.43 as well as no significant differences between the motion during task and resting conditions at *p* ≥ 0.94. Following spatial smoothing of EPI data with a 4-mm Gaussian filter, a single regressor for each task was convolved with a canonical hemodynamic response function and entered into a multiple regression model to predict the observed BOLD response. To correct for residual head motions, six motion parameter estimates were included as covariates of no interest, and quadratic polynomials in time were used to model baseline drifts for each imaging run. Following spatial normalization, a one-way analysis of variance was used for group-level statistical analysis to assess (1) the effect of SXB on abnormal brain function by measuring differences in neural activity before vs. after SXB intake in each patient group, separately, and compared to healthy controls, and (2) modulation of abnormal brain activity by SXB and botulinum toxin. As an exploratory study, we further used a two-sample independent *t*-test to examine the differences between SXB+ and SXB− patients. Statistical significance was set at a family-wise (FWE) corrected *p* ≤ 0.05 in AFNI software by randomizing and permuting input datasets to simulate the noise volumes and control the false positive rate. Correction for multiple comparisons at a cluster level was performed to ensure that each reported region reached statistical significance at *a priori p* ≤ 0.01.

### Clinical correlations

The ratings of voice symptoms at a baseline and 40 min after drug intake were used for correlations between SXB effects on voice symptoms and brain activation in LD and LD/DTv patients, separately. Using AFNI software, voxelwise Spearman’s rank correlation coefficients were computed to assess the statistical dependence of post-drug mean percent BOLD signal change during symptomatic speech production with LD and DTv symptoms, respectively, at FWE-corrected *p* ≤ 0.05.

## Data Availability

The datasets generated during the current study are available from the corresponding author on reasonable request.

## References

[1] Kirke D. N. *et al*. Neural correlates of dystonic tremor: a multimodal study of voice tremor in spasmodic dysphonia. *Brain Imaging Behav*. Feb 3. [Epub ahead of print] (2016). 10.1007/s11682-016-9513-x PMC4972702 26843004

[2] Blitzer A (2010). Spasmodic dysphonia and botulinum toxin: experience from the largest treatment series. Eur J Neurol..

[3] Novakovic D, Waters HH, D’Elia JB, Blitzer A (2011). Botulinum toxin treatment of adductor spasmodic dysphonia: longitudinal functional outcomes. Laryngoscope..

[4] Pirio Richardson S (2017). Dystonia treatment: Patterns of medication use in an international cohort. Neurology..

[5] Kirke DN, Frucht SJ, Simonyan K (2015). Alcohol responsiveness in laryngeal dystonia: a survey study. J Neurol..

[6] Nestoros JN (1980). Ethanol specifically potentiates GABA-mediated neurotransmission in feline cerebral cortex. Science..

[7] Hallett M (2011). Neurophysiology of dystonia: The role of inhibition. Neurobiol Dis..

[8] Levy LM, Hallett M (2002). Impaired brain GABA in focal dystonia. Ann Neurol..

[9] Quartarone A, Hallett M (2013). Emerging concepts in the physiological basis of dystonia. Mov Disord..

[10] Simonyan, K. Inferior parietal cortex as a hub of loss of inhibutuib and maladaptive plasticity. Paper presented at: Annual Meeting of Americal ACademy of Neurology; Boston (2017).

[11] Rumbach, A. F., Blitzer, A., Frucht, S. J. & Simonyan, K. An open-label study of sodium oxybate in Spasmodic dysphonia. *Laryngoscope*. 2016. 10.1002/lary.26381 PMC5415435 27808415

[12] Simonyan, K. & Frucht, S. J. Long-term Effect of Sodium Oxybate (Xyrem(R)) in Spasmodic Dysphonia with Vocal Tremor. *Tremor and other hyperkinetic movements*. 3. eCollection 2013 (2013) 10.7916/D8CJ8C5S PMC3863985 24386608

[13] Waszkielewicz A, Bojarski J (2004). Gamma-hydrobutyric acid (GHB) and its chemical modifications: a review of the GHBergic system. Pol J Pharmacol..

[14] Wang YG, Swick TJ, Carter LP, Thorpy MJ, Benowitz NL (2009). Safety overview of postmarketing and clinical experience of sodium oxybate (Xyrem): abuse, misuse, dependence, and diversion. J Clin Sleep Med..

[15] Ali SO (2006). Alterations in CNS activity induced by botulinum toxin treatment in spasmodic dysphonia: an H215O PET study. J Speech Lang Hear Res..

[16] Battistella, G., Fuertinger, S., Fleysher, L., Ozelius, L. J. & Simonyan, K. Cortical sensorimotor alterations classify clinical phenotype and putative genotype of spasmodic dysphonia. *European journal of neurology*. (2016). 10.1111/ene.13067 PMC5308055 27346568

[17] Haslinger B (2005). “Silent event-related” fMRI reveals reduced sensorimotor activation in laryngeal dystonia. Neurology..

[18] Simonyan K, Ludlow CL (2010). Abnormal activation of the primary somatosensory cortex in spasmodic dysphonia: an fMRI study. Cereb Cortex..

[19] Battistella, G., Termsarasab, P., Ramdhani, R. A., Fuertinger, S. & Simonyan, K. Isolated Focal Dystonia as a Disorder of Large-Scale Functional Networks. *Cereb Cortex*. pii: bhv313. [Epub ahead of print] (2015) 10.1093/cercor/bhv313 PMC6075177 26679193

[20] Lehericy S, Tijssen MA, Vidailhet M, Kaji R, Meunier S (2013). The anatomical basis of dystonia: current view using neuroimaging. Mov Disord..

[21] Zoons E, Booij J, Nederveen AJ, Dijk JM, Tijssen MA (2011). Structural, functional and molecular imaging of the brain in primary focal dystonia–a review. Neuroimage..

[22] Simonyan K, Ludlow CL (2012). Abnormal structure-function relationship in spasmodic dysphonia. Cereb Cortex..

[23] Simonyan K (2008). Focal white matter changes in spasmodic dysphonia: a combined diffusion tensor imaging and neuropathological study. Brain..

[24] Ramdhani RA (2014). What’s special about task in dystonia? A voxel-based morphometry and diffusion weighted imaging study. Mov Disord..

[25] Kostic VS (2016). Brain structural changes in spasmodic dysphonia: A multimodal magnetic resonance imaging study. Parkinsonism Relat Disord..

[26] Prudente CN (2013). Neuropathology of cervical dystonia. Exp Neurol..

[27] Kulisevsky J, Marti MJ, Ferrer I, Tolosa E (1988). Meige syndrome: neuropathology of a case. Mov Disord..

[28] Hoshi E, Tremblay L, Feger J, Carras PL, Strick PL (2005). The cerebellum communicates with the basal ganglia. Nat Neurosci..

[29] Bostan AC, Dum RP, Strick PL (2010). The basal ganglia communicate with the cerebellum. Proc Natl Acad Sci USA.

[30] Middleton FA, Strick PL (2000). Basal ganglia and cerebellar loops: motor and cognitive circuits. Brain Res Brain Res Rev..

[31] Dum RP, Li C, Strick PL (2002). Motor and nonmotor domains in the monkey dentate. Ann N Y Acad Sci..

[32] Brighina F (2009). Effects of cerebellar TMS on motor cortex of patients with focal dystonia: a preliminary report. Exp Brain Res..

[33] Chen CH, Fremont R, Arteaga-Bracho EE, Khodakhah K (2014). Short latency cerebellar modulation of the basal ganglia. Nat Neurosci..

[34] Garibotto V (2011). *In vivo* evidence for GABA(A) receptor changes in the sensorimotor system in primary dystonia. Mov Disord..

[35] Hallett M (2004). Dystonia: abnormal movements result from loss of inhibition. Adv Neurol..

[36] Crunelli V, Emri Z, Leresche N (2006). Unravelling the brain targets of gamma-hydroxybutyric acid. Curr Opin Pharmacol..

[37] Absalom N (2012). alpha4betadelta GABA(A) receptors are high-affinity targets for gamma-hydroxybutyric acid (GHB). Proc Natl Acad Sci USA.

[38] Ludlow CL (2008). Research priorities in spasmodic dysphonia. Otolaryngol Head Neck Surg..

[39] Arpesella R (2009). A patient with intractable posthypoxic myoclonus (Lance-Adams syndrome) treated with sodium oxybate. Anaesthesia and intensive care..

[40] Frucht SJ, Bordelon Y, Houghton WH, Reardan D (2005). A pilot tolerability and efficacy trial of sodium oxybate in ethanol-responsive movement disorders. Mov Disord..

[41] Frucht SJ, Houghton WC, Bordelon Y, Greene PE, Louis ED (2005). A single-blind, open-label trial of sodium oxybate for myoclonus and essential tremor. Neurology..

[42] Simonyan K, Berman BD, Herscovitch P, Hallett M (2013). Abnormal striatal dopaminergic neurotransmission during rest and task production in spasmodic dysphonia. J Neurosci..

